# Circ_0008035 Promotes Gastric Cancer Development via the miR-429/SMAD2 Cascade

**DOI:** 10.5152/tjg.2024.23341

**Published:** 2024-10-01

**Authors:** Yan Chen, Weigang Bian, Surong Chen

**Affiliations:** 1 Department of Medical Oncology, Yancheng First People’s Hospital, Yancheng, Jiangsu, China

**Keywords:** circ_0008035, gastric cancer, miR-429, SMAD2

## Abstract

**Background/Aims:**

The vital roles of circular RNAs (circRNAs) in human tumorigenesis have attracted more attention. Circ_0008035 is one of the most up-regulated circRNAs in gastric cancer (GC). Herein, we explored the associated mechanism of circ_0008035 in GC.

**Materials and Methods:**

EdU incorporation experiments were performed to monitor cell proliferation ability. Cell cycle progression, apoptosis, angiogenesis, migration, and invasion were analyzed using flow cytometry, Tube formation, and Transwell assays respectively. Protein expression was detected by Western blot. Dual-luciferase reporter experiments were applied to demonstrate the relationship between circ_0008035 or SMAD family member 2 (SMAD2) and microRNA-429 (miR-429). Mouse xenograft assays were conducted for evaluation of the role of circ_0008035 in vivo.

**Results:**

Circ_0008035 content was elevated in GC tissues (*P *< .0001) and cell lines (*P *< .001), and its deficiency hindered GC cell proliferation (*P *< .01), HUVEC angiogenesis (*P *< .05), and GC cell metastasis (*P *< .01) and triggered apoptosis (*P *< .01). Circ_0008035 could sponge miR-429 to up-regulate SMAD2 expression (*P *< .0001). Circ_0008035 absence restrained tumor growth in vivo (*P *< .01). MiR-429 was a mediator of circ_0008035 function, and miR-429 hindered GC cell malignant phenotypes by SMAD2.

**Conclusion:**

Circ_0008035 aggravates GC cell malignant progression partially by targeting the miR-429/SMAD2 axis. Considering the inhibitory effect of circ_0008035 deficiency on GC progression, targeting circ_0008035 may be a potential approach to prevent or treat GC.

**Main Points**

- Circ_0008035 absence suppresses GC cell progression.
- miR-429 is a direct target of circ_0008035.
- miR-429 directly interacts with the 3’UTR of SMAD2.

## Introduction

Gastric cancer (GC) is one of the most deadly malignancies worldwide. In recent years, the advancement of diagnostic and therapeutic strategies has greatly improved the incidence and mortality of GC.^1,2^ Nevertheless, owing to tumor metastasis and recurrence, the 5-year survival rate for GC cases is still dismal.^3^ Therefore, exploring the mechanisms underlying GC pathogenesis is essential to develop effective GC treatment strategies.

Numerous circular RNAs (circRNAs) partake in tumorigenesis through modulation of cell phenotypes, including cell growth, motility, and apoptosis.^4,5^ As an example, circ-PRMT5 aggravates breast cancer progression by targeting the microRNA-509-3p (miR-509-3p)/TCF7L2 cascade and activating the PI3K/AKT pathway.^6^ Circ-DONSON can contribute to the growth and metastasis of GC cells via NURF complex-dependent activation of SOX4.^7^ Based on the data of the GSE78092 dataset, circ_0008035 is found to be the most significantly up-regulated circRNA in GC tissue specimens versus normal gastric tissues.^8^ Nevertheless, its role in GC remains to be clarified.

Convincing evidence indicates that circRNAs can sequester microRNAs (miRNAs) to reduce their activity.^9,10^ Furthermore, miRNAs can post-transcriptionally silence their target genes by binding to the 3’UTR of mRNAs.^11^ Herein, the expression and role of circ_0008035 were assessed in GC cell lines. Then, interacting molecules of circ_0008035 were explored using the starBase tool, and its mechanism was verified via further functional assays.

## Materials and Methods

### Clinical Study

GC and adjacent normal specimens from 31 GC sufferers were harvested from Yancheng First People’s Hospital. The procedure was authorized by the Ethics Committee of Yancheng First People’s Hospital (approval no: 2020-9, date: September 21, 2020). All GC cases signed written informed consent and did not receive pre-operative anti-cancer therapy.

### Cell Culture

Human AGS, HGC-27, and GES-1 cell lines (Cell Resource Center of the Chinese Academy of Science, Shanghai, China) were maintained in DMEM enriched with 10% FBS (Gibco, Carlsbad, Calif, USA) in a humidified incubator containing 5% CO_2_ at 37°C.

### RT-qPCR

For miR-429 analysis, cDNA was synthesized using a stem-loop primer (Ribobio, Guangzhou, China) and was amplified via a miRNA-specific forward primer and universal reverse primer. Reverse transcription for circ_0008035, exostosin glycosyltransferase 1 (EXT1), and SMAD family member 2 (SMAD2) was performed using TaqMan Reagents (Invitrogen, Waltham, MA, USA). Subsequently, RNA expression content was tested by qPCR with a SYBR Green Kit (Takara, Dalian, China) and specific primers listed in Table 1. The 2^-ΔΔCt^ method was used to analyze the results.

### Cell Transfection

Circ_0008035-specific siRNA (si-circ_0008035), si-NC, sh-circ_0008035, sh-NC, pLCDH-circ_0008035 plasmid (oe-circ_0008035), oe-NC, miR-429 mimic, miR-NC mimic, miR-429 inhibitor, miR-NC inhibitor, pcDNA-SMAD2 vector (pcDNA-SMAD2), and pcDNA-NC were provided by Genepharma (Shanghai, China). Transfection of plasmids was implemented via X-tremeGENE HP Reagent (Roche, Shanghai, China), and transfection of RNA oligonucleotides was performed based on Lipofectamine 2000 (Invitrogen).

### EdU Incorporation Assay

For proliferation analysis, GC cells were maintained in media containing 50 μM EdU (RiboBio) for 2 hours, and then subjected to staining with Apollo Dye Solution (RiboBio). Then, cell nuclei were subjected to DAPI staining, followed by analysis of the EdU-positive cells under a fluorescence microscope.

### Flow Cytometry

For cell cycle progression analysis, cells grown in the culture dishes were collected by trypsinization and then immobilized with 80% ethanol overnight. Bovine pancreatic RNase (Sigma) was applied to remove RNA. Cells were then incubated with 20 mg/mL of PI (Sigma) for 20 minutes. The cell cycle phase was evaluated.

To evaluate cell apoptosis, collected GC cells were dispersed in binding buffer and mixed with Annexin V and PI (BD Biosciences, Heidelberg, Germany) for 15 minutes. Cell apoptosis was analyzed.

### Tube Formation Assay

HUVECs were suspended in the conditioned culture medium obtained from treated GC cells. After 8 hours, cell angiogenesis was analyzed by counting the number of formed tubes in 5 random fields using a microscope (Olympus).

### Transwell Assay

Transwell chambers with Matrigel-precoated (Yeasen Biotechnology, Shanghai, China) inserts were used to evaluate cell invasion. Uncoated upper compartments were used for evaluation of cell migration ability. Transwell compartments were placed into 24-well plates. Cells were resuspended in non-serum medium and added to the Matrigel-coated or uncoated upper compartments. The lower compartments were supplemented with 600 μL of 20% FBS DMEM. Migrated or invaded GC cells were stained with 0.5% crystal violet (Sigma). The number of migrated or invaded GC cells was counted using an inverted optical microscope (Leica, Wetzlar, Germany).

### Western Blot Assay

After being separated by 10% SDS-PAGE, protein samples were blotted onto PVDF membranes, which were then hatched with the following primary antibodies overnight (Cell Signaling Technology, Boston, Mass, USA): anti-E-cadherin (#14472), anti-vimentin (#5741), anti-SMAD2 (#5339), and anti-β-actin (#8457). After incubation with the secondary antibody (Cell Signaling Technology), bands were developed via the ECL kit as described by the producer (Beyotime, Shanghai, China).

### Dual-Luciferase Reporter Assay

To generate circ_0008035 reporter constructs, the circ_0008035 fragment harboring the wild-type (WT) predicted complementary sequence for miR-429, or mutations (MUT) in the binding sequence was inserted into the pmirGLO vector. To generate SMAD2 3’UTR reporter constructs, the SMAD2 3’UTR fragment harboring the WT binding sequence for miR-429 or its mutations (MUT) was cloned into the pmirGLO vector. After co-transfection with small RNAs and plasmids, luciferase intensities were examined using a commercial kit (Promega, Madison, WI, USA).

### Xenograft Tumor Model

AGS cells infected with sh-NC or sh-circ_0008035 lentivirus were subcutaneously injected into male nude mice (4-6 weeks old, n = 5 for each group, Vital River Laboratory, Beijing, China). Tumor size was measured every week, and xenograft volume was calculated using the method (length × width^2^ × 0.5). Four weeks later, xenograft tumors from euthanized mice were excised and weighed. These assays were authorized by the Ethics Committee of Animal Research of Yancheng First People’s Hospital.

### Statistical Analysis

Difference analysis (*P *< .05) was done using Student’s *t*-test or one-way ANOVA. Expression association was evaluated using Pearson’s correlation coefficient. All data in the current research were exhibited as mean ± SD.

## Results

### Circ_0008035 Level Is Aberrantly Increased in GC

**Figure 1.**
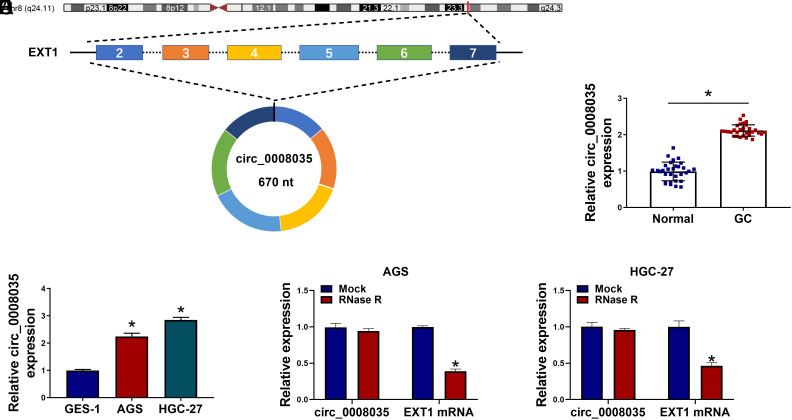
Circ_0008035 level is aberrantly up-regulated in GC tissues and cell lines. (A) Circ_0008035 consists of 6 exons (exon 2-7, 670 nt) which are derived from its host gene EXT1. (B) RT-qPCR was conducted to determine the expression of circ_0008035 in 31 pairs of GC tissues and normal adjacent tissues. (C) The level of circ_0008035 was examined in GC cell lines (AGS and HGC-27) and the human gastric mucosa cell line (GES-1) by RT-qPCR. (D and E) Total RNA samples were treated with or without RNase R, and the levels of circ_0008035 and its linear counterpart EXT1 mRNA were examined by RT-qPCR. *P < .05.

Circ_0008035, originating from exons 2 to 7 of the pre-mRNA of the host gene EXT1, is a circular RNA molecule with a length of 670 nucleotides (nt) (Figure 1A). Circ_0008035 was markedly enhanced in GC tissues and GC cell lines (AGS and HGC-27) relative to their corresponding controls (Figure 1B and C). Moreover, RNase R treatment showed that the linear EXT1 mRNA, rather than circ_0008035, could be degraded by RNase R (Figure 1D and E), indicating that circ_0008035 has a higher tolerance to exonuclease.

### Circ_0008035 Knockdown Hampers GC Cell Malignant Behaviors

**Figure 2.**
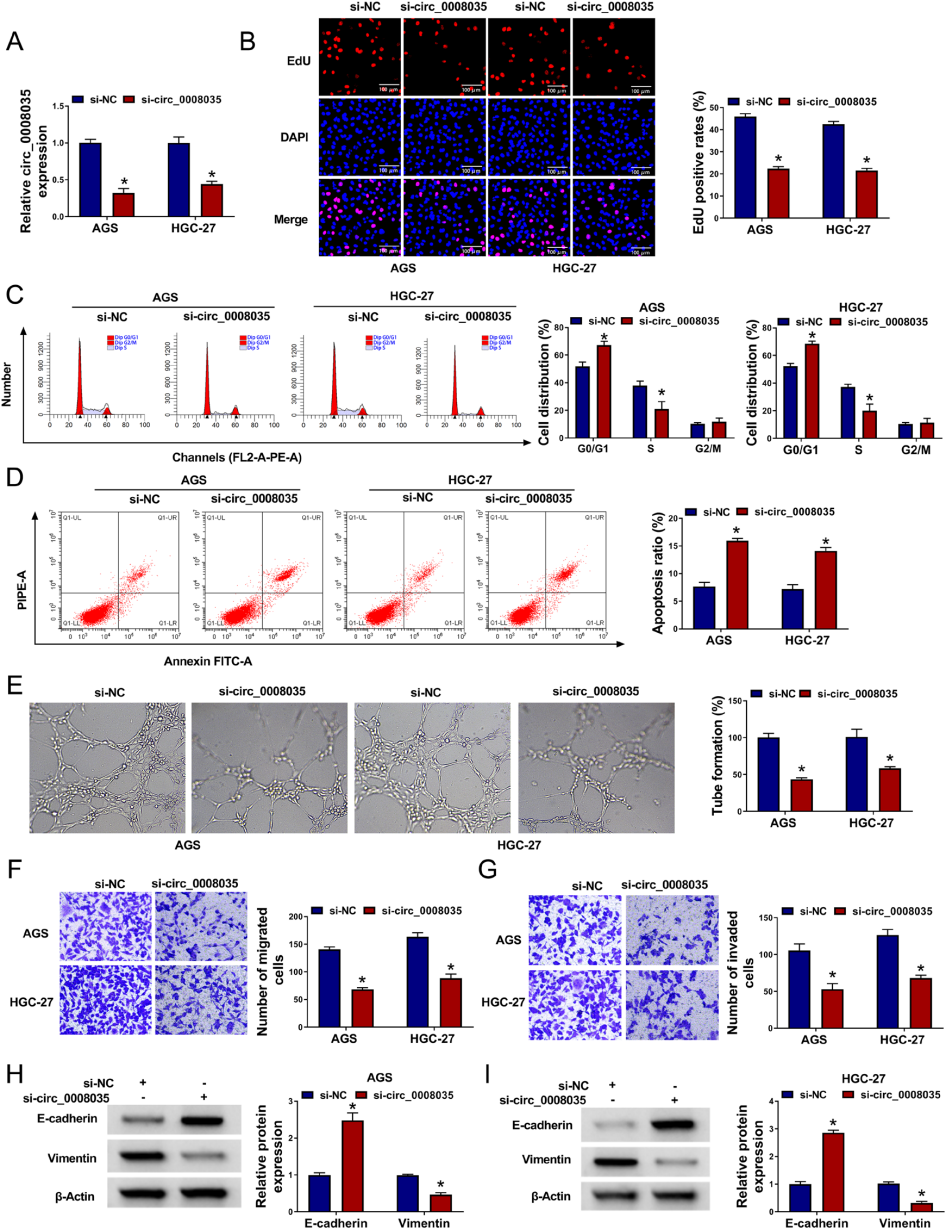
Circ_0008035 knockdown hampers the proliferation, angiogenesis, migration, and invasion and facilitates the apoptosis of GC cells. (A-I) GC cells were transfected with si-NC or si-circ_0008035. (A) RT-qPCR was conducted to analyze the expression of circ_0008035 in transfected GC cells. (B) EdU incorporation assay was conducted to analyze the proliferation ability of transfected GC cells. (C and D) Cell cycle progression and apoptosis were analyzed by flow cytometry. (E) Tube formation assay was performed to assess the angiogenesis ability of transfected GC cells. (F and G) Cell migration and invasion abilities were evaluated by transwell assays. (H and I) Western blot assay was performed to detect the protein levels of E-cadherin and vimentin in transfected GC cells. *P < .05.

Subsequently, knockdown efficiency of the circ_0008035 was validated in GC cells (Figure 2A). Functionally, circ_0008035 depletion reduced the percentage of EdU-positive GC cells (Figure 2B), suggesting that circ_0008035 deficiency blocked cell proliferation. Circ_0008035 absence also reduced the number of cells in the S stage (Figure 2C), indicating that circ_0008035 deficiency repressed cell cycle progression. Conversely, cell apoptosis was elevated by circ_0008035 knockdown (Figure 2D). Circ_0008035 silencing suppressed the tube formation of HUVECs (Figure 2E). Additionally, circ_0008035 absence resulted in suppressed migration and invasion abilities of GC cells (Figure 2F and G). Western blot assay was conducted to analyze the expression of 2 EMT-associated proteins (E-cadherin and vimentin) in transfected GC cells. Circ_0008035 absence increased the expression of E-cadherin and reduced the expression of vimentin (Figure 2H and I).

### Circ_0008035 Acts as a Molecular Sponge for miR-429

**Figure 3.**
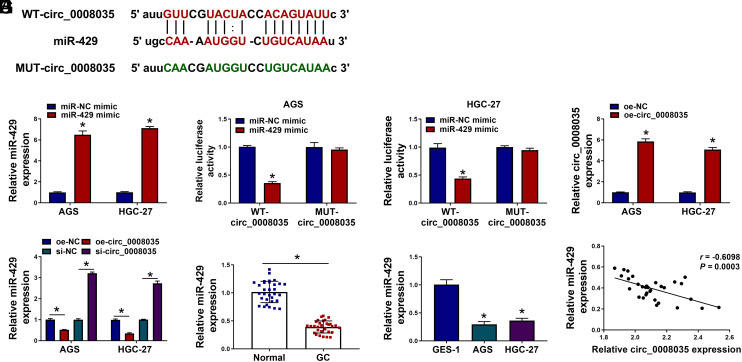
Circ_0008035 acts as a molecular sponge for miR-429. (A) The interacting miRNAs of circ_0008035 were predicted by the starBase database, and miR-429 was one of the possible targets. (B) RT-qPCR was conducted to analyze the overexpression efficiency of the miR-429 mimic in GC cells. (C) Dual-luciferase reporter assay was performed to verify the interaction between circ_0008035 and miR-429 in GC cells. (D) The overexpression efficiency of the circ_0008035 plasmid (oe-circ_0008035) in GC cells was evaluated by RT-qPCR. (E) GC cells were transfected with oe-NC, oe-circ_0008035, si-NC, or si-circ_0008035, and the level of miR-429 was determined in transfected GC cells by RT-qPCR. (F) RT-qPCR was carried out to examine the expression of miR-429 in 31 pairs of GC tissues and normal adjacent tissues. (G) The level of miR-429 in GES-1, AGS, and HGC-27 cells was analyzed by RT-qPCR. (H) Pearson’s correlation analysis was conducted to generate the linear correlation between the levels of circ_0008035 and miR-429 in GC tissues. *P < .05.

CircRNAs can function as miRNA sponges to modulate cancer cell biological behaviors.^9,10^ We predicted the interacting miRNAs of circ_0008035 based on the starBase database. Results showed that circ_0008035 harbored a potential binding sequence for miR-429 (Figure 3A). Overexpression efficiency of miR-429 was demonstrated and displayed in Figure 3B. Enhanced miR-429 expression reduced the luciferase activity of cells transfected with WT-circ_0008035, but not MUT-circ_0008035 (Figure 3C), implying the direct interaction between circ_0008035 and miR-429. Overexpression efficiency of the circ_0008035 was shown in Figure 3D. Circ_0008035 overexpression reduced miR-429 levels, while circ_0008035 silencing up-regulated miR-429 expression in GC cells (Figure 3E). Moreover, miR-429 content was down-regulated in GC tissues and cells (Figure 3F and G), and inversely associated with circ_0008035 content in GC tumors (Figure 3H). All these observations indicate that circ_0008035 directly targets miR-429.

### Reduction of miR-429 Partially Counteracts the Effects of Circ_0008035 Silencing in GC Cells

**Figure 4.**
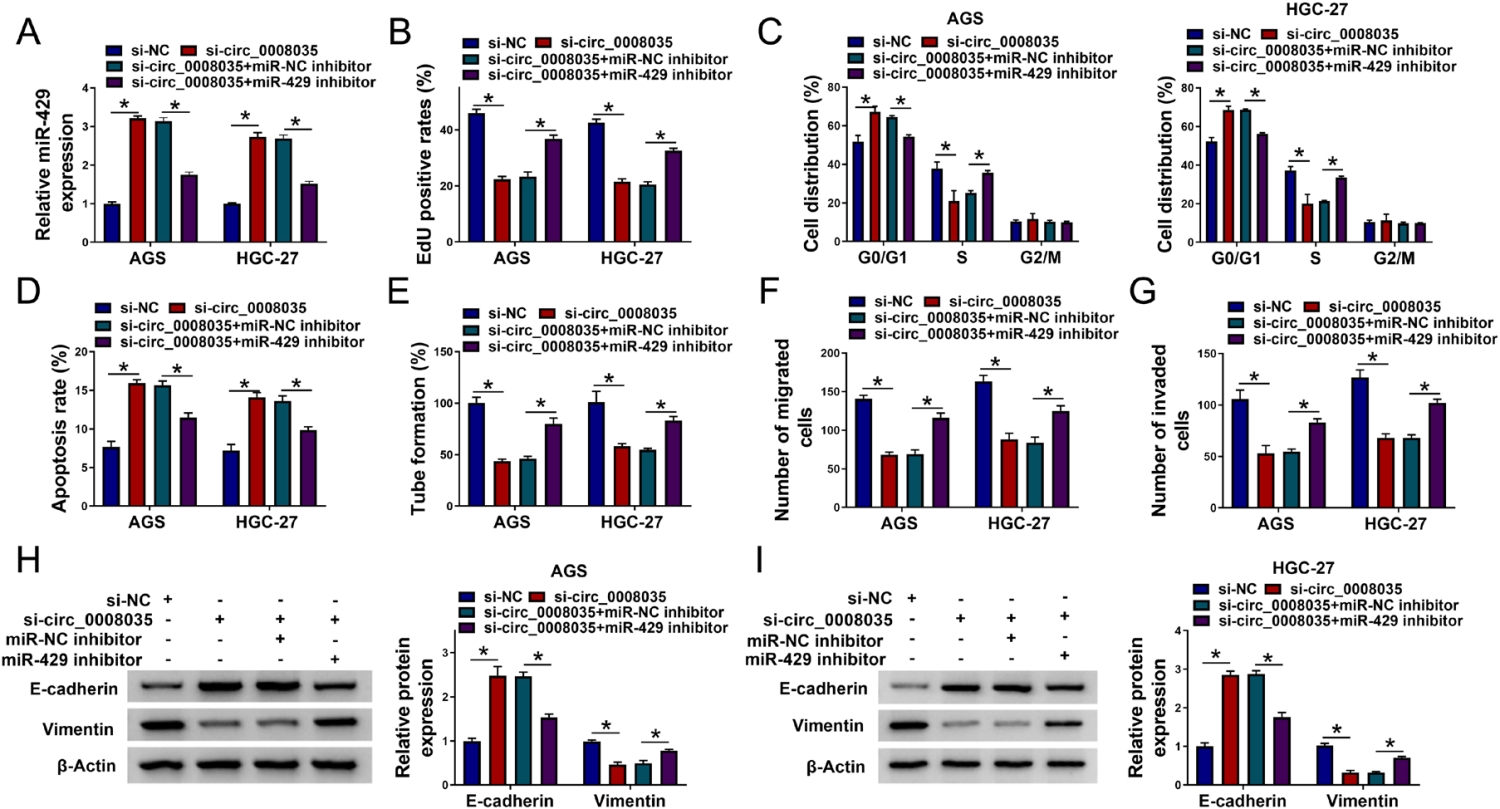
The effects induced by circ_0008035 knockdown in GC cells can be largely counteracted by the silencing miR-429. (A-I) GC cells were transfected with si-circ_0008035 alone or together with the miR-429 inhibitor. (A) The level of miR-429 in GC cells was determined by RT-qPCR. (B) Cell proliferation was analyzed by EdU incorporation assay. (C) Cell cycle progression was analyzed by flow cytometry. (D) Cell apoptosis was analyzed by flow cytometry. (E) The angiogenesis ability of GC cells was analyzed by tube formation assay. (F and G) Transwell assays were conducted to analyze the migration and invasion abilities of GC cells. (H and I) The protein levels of E-cadherin and vimentin were determined by western blot assay. *P < .05.

Then, we performed rescue experiments in GC cells. Circ_0008035 knockdown up-regulated miR-429 content, and the miR-429 inhibitor reversed the impact (Figure 4A). Circ_0008035 knockdown-imposed proliferation repression and apoptosis promotion were significantly counteracted by the silencing of miR-429 (Figure 4B-D). MiR-429 downregulation also reversed circ_0008035 deficiency-driven suppression in HUVEC tube formation and repression in GC cell migration and invasion (Figure 4E-G). Moreover, the miR-429 inhibitor clearly abated the circ_0008035 deficiency-mediated E-cadherin expression increase and vimentin level reduction (Figure 4H and I). Overall, we conclude that the circ_0008035/miR-429 cascade regulates GC progression in vitro.

### miR-429 Interacts with the 3’UTR of SMAD2

**Figure 5.**
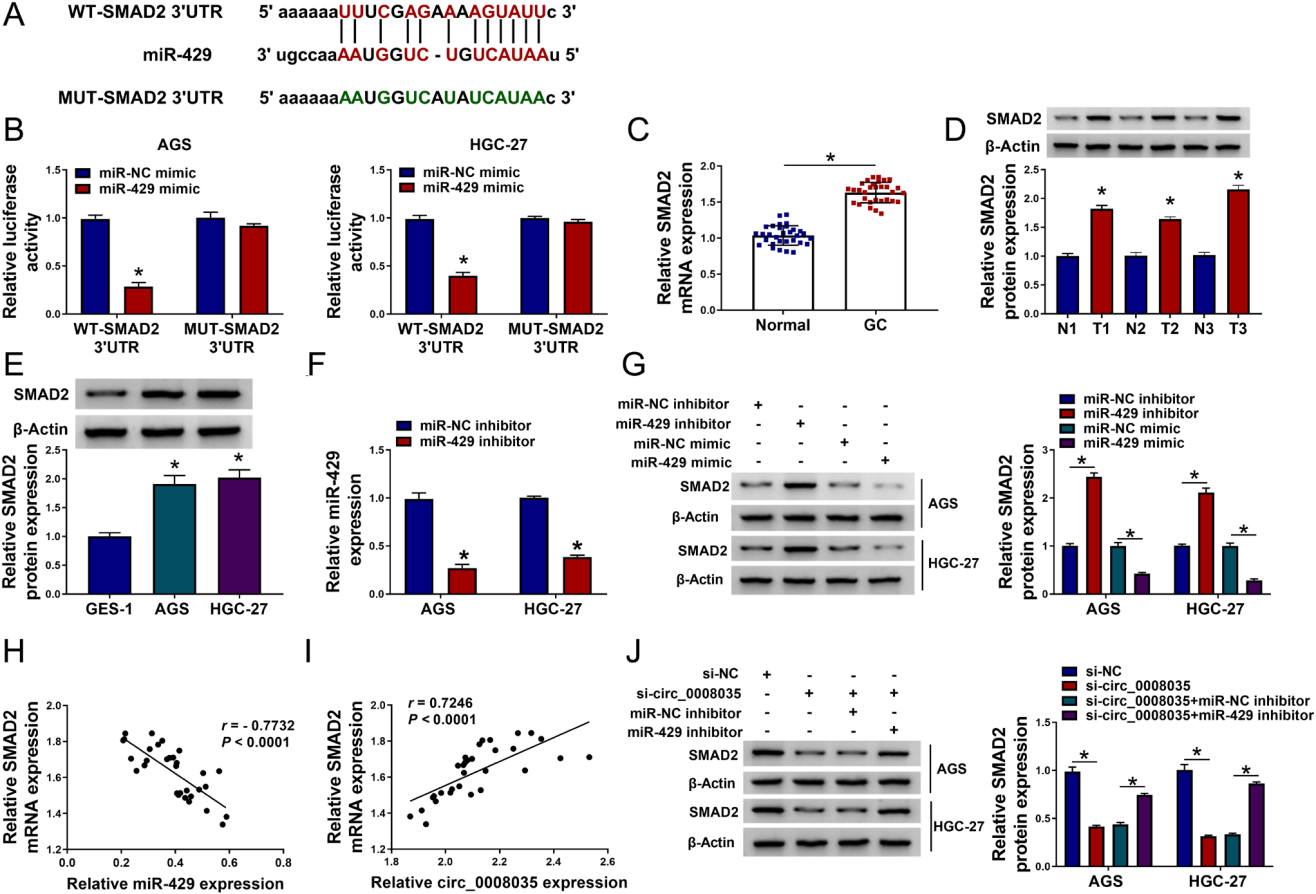
miR-429 interacts with the 3’UTR of SMAD2. (A) SMAD2 was predicted as one of the possible targets of miR-429 by the starBase database. (B) The interaction between miR-429 and SMAD2 was confirmed by a dual-luciferase reporter assay. (C and D) RT-qPCR and western blot assays were conducted to determine the mRNA and protein levels of SMAD2 in GC tissues and adjacent normal tissues. (E) Western blot assay was carried out to detect the protein expression of SMAD2 in GC cell lines and the GES-1 cell line. (F) The knockdown efficiency of the miR-429 inhibitor in GC cells was analyzed by RT-qPCR. (G) GC cells were transfected with miR-NC inhibitor, miR-429 inhibitor, miR-NC mimic, or miR-429 mimic. The protein expression of SMAD2 in transfected GC cells was examined by western blot assay. (H and I) The linear correlation between the expression of SMAD2 mRNA and miR-429 or circ_0008035 in GC tissues was evaluated by Pearson’s correlation analysis. (J) GC cells were transfected with si-circ_0008035 alone or together with the miR-429 inhibitor, and the protein level of SMAD2 was examined by western blot assay. *P < .05.

MiRNAs control gene expression by directly binding to mRNA 3’UTR.^11^ Through the starBase tool, we found that SMAD2 was a possible target of miR-429 (Figure 5A). miR-429 overexpression significantly diminished the luciferase activity of cells transfected with WT-SMAD2 3’UTR, but not MUT-SMAD2 3’UTR (Figure 5B). SMAD2 content was elevated in GC tissues (Figure 5C and D) and GC cell lines (AGS and HGC-27) (Figure 5E). Knockdown efficiency of the miR-429 inhibitor is shown in Figure 5F. SMAD2 protein level was up-regulated by miR-429 silencing and diminished by miR-429 upregulation (Figure 5G). In GC tissues, SMAD2 mRNA expression was inversely correlated with miR-429 level and positively associated with circ_0008035 expression (Figure 5H and I). Furthermore, down-regulation of miR-429 by introduction of an miR-429 inhibitor abated the circ_0008035 absence-mediated decrease of SMAD2 protein expression in GC cells (Figure 5J). Together, these findings suggest that circ_0008035 increases SMAD2 content by sponging miR-429 in GC cells.

### miR-429 Represses GC Cell Development Via SMAD2

**Figure 6.**
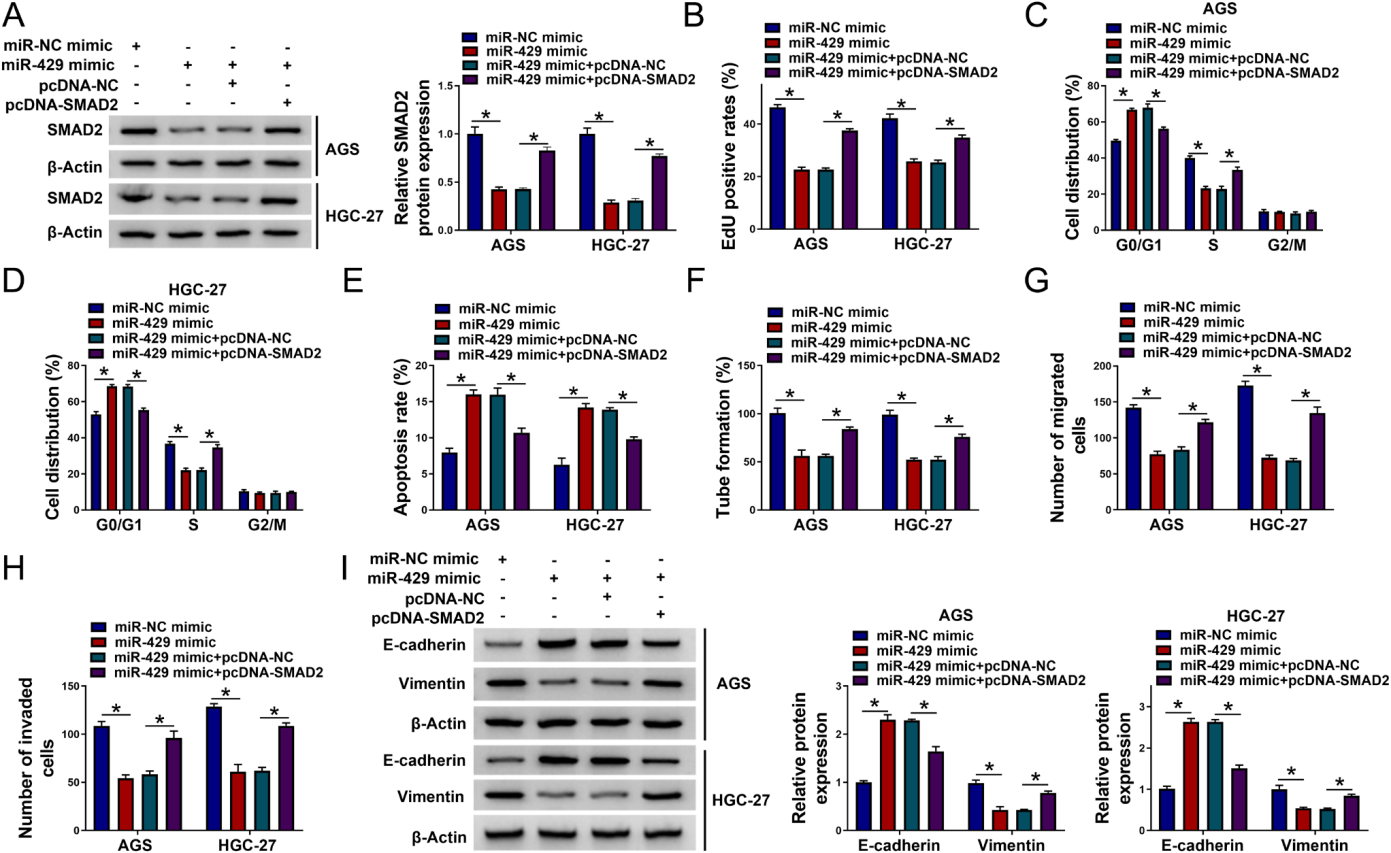
miR-429 overexpression-mediated effects in GC cells are largely overturned by the overexpression of SMAD2. (A-I) GC cells were transfected with miR-429 mimic alone or together with pcDNA-SMAD2. (A) A western blot assay was conducted to determine the expression of the SMAD2 protein in transfected GC cells. (B-D) Cell proliferation was analyzed by EdU incorporation assay and flow cytometry. (E) Flow cytometry was conducted to analyze the apoptosis of GC cells. (F) A tube formation assay was carried out to analyze the angiogenesis ability of GC cells. (G and H) Transwell assays were implemented to analyze the migration and invasion abilities of GC cells. (I) The protein levels of E-cadherin and vimentin were examined by western blot assay. *P < .05.

We next wondered whether miR-429 regulates GC progression by targeting SMAD2. Elevated miR-429 levels reduced SMAD2 protein expression, which was restored by pcDNA-SMAD2 introduction (Figure 6A). Functional experiments revealed that miR-429 overexpression suppressed cell proliferation, repressed cell cycle progression, and promoted cell apoptosis in GC cells; these effects were significantly abrogated by SMAD2 upregulation (Figure 6B-E). Overexpression of miR-429 also reduced cell tube formation, migration, and invasion abilities; SMAD2 upregulation partly abolished these effects (Figure 6F-H). In addition, SMAD2 upregulation abated miR-429 overexpression-imposed alterations of E-cadherin and vimentin levels (Figure 6I). Taken together, we conclude that miR-429 overexpression suppresses GC progression by down-regulating SMAD2.

### Circ_0008035 Absence Significantly Diminishes the Growth of Xenograft Tumors In Vivo

**Figure 7.**
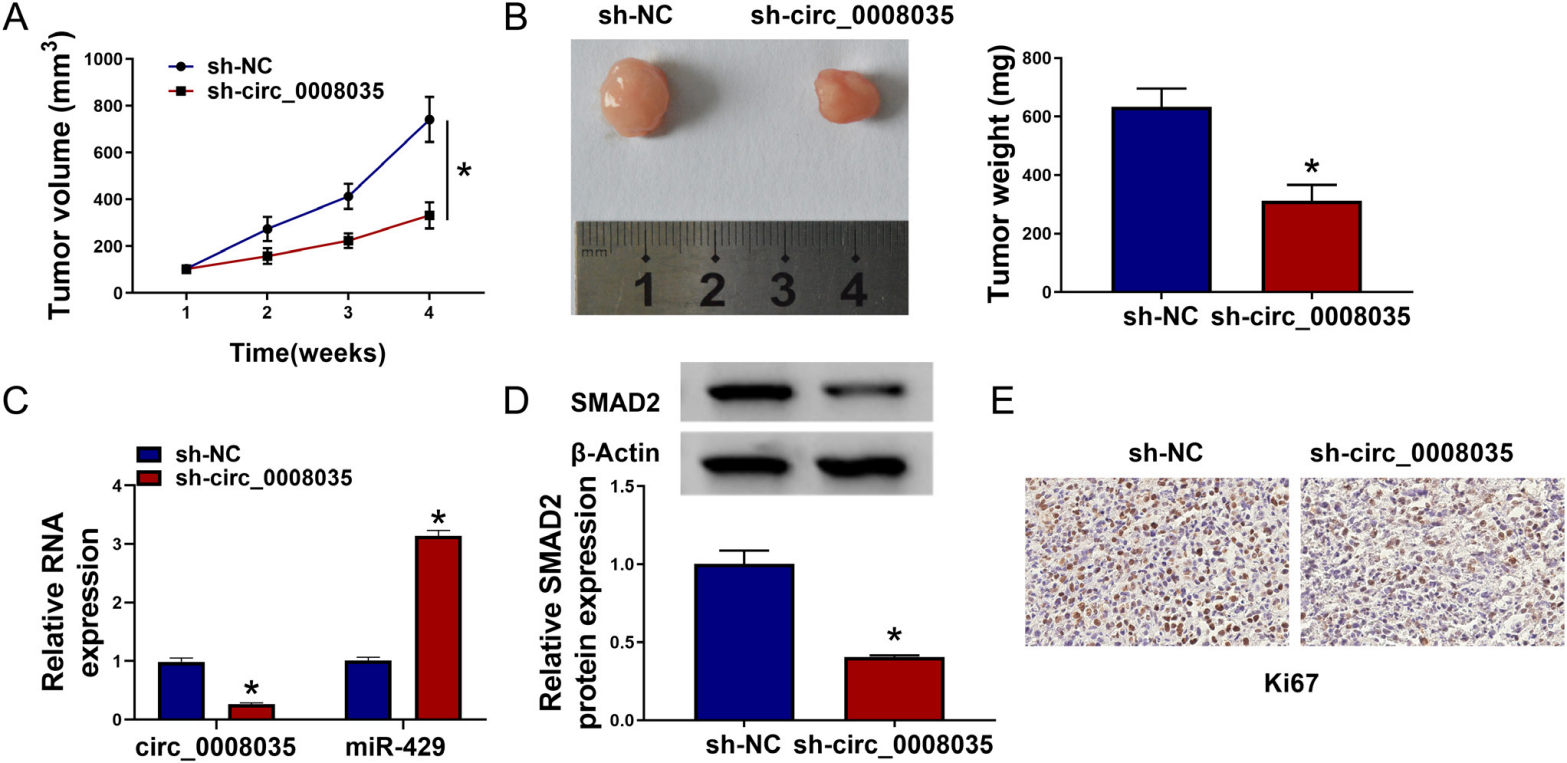
Circ_0008035 knockdown significantly blocks tumor progression in vivo. (A) Tumor length and width were measured after subcutaneous injection every week, and tumor volume was analyzed as length × width^2^ × 0.5. (B) After injection for 4 weeks, all xenograft tumors were excised and weighed. (C) RT-qPCR was conducted to analyze the expression of circ_0008035 and miR-429 in tumor tissues. (D) Western blot assay was performed to determine the protein expression of SMAD2 in tumor tissues. (E) IHC assay was conducted to detect the protein level of Ki67 in tumor tissues. *P < .05.

Additionally, we found that circ_0008035 knockdown suppressed tumor growth in vivo compared with the sh-NC group (Figure 7A and B). Circ_0008035 and SMAD2 expression levels were reduced, and miR-429 was increased in sh-circ_0008035 xenografts (Figure 7C and D). IHC results validated that the number of Ki67-positive cells was reduced in sh-circ_0008035 xenografts (Figure 7E). Overall, these results indicate that circ_0008035 depletion suppresses the growth of xenograft tumors in vivo.

## Discussion

CircRNAs have been implicated in the development of human malignant tumors, including GC.^12^ Through the circRNA microarray technique, circ_0008035 is found to be one of the most significantly up-regulated circRNAs in GC specimens.^8^ Our data uncover that circ_0008035 depletion restrains tumor cell growth and metastasis, implying the oncogenic activity of circ_0008035 in GC, in line with previous reports.^13, 14^ Importantly, we provide a molecular determinant for circ_0008035’s oncogenic activity via a novel miRNA/mRNA cascade. This study provides novel molecular evidence for developing circ_0008035 as a possible therapeutic target against GC.

Emerging laboratory work has discovered the role of circRNAs as miRNA “sponges.”^9^ CircRNAs can function as miRNA inhibitors to negatively modulate miRNA activity, thereby promoting downstream target activity.^15^ For instance, circ_100395 increases TCF21 expression to suppress lung cancer development by sequestering miR-1228.^16^ Circ_0000515 can aggravate cervical cancer progression by targeting the miR-326/ELK1 axis.^17^ Here, we demonstrate that circ_0008035 directly targets miR-429. miR-429 restrains breast cancer cell growth and invasion by inactivating the Wnt/β-catenin cascade.^18^ miR-429 hampers thyroid cancer cell growth and facilitates cell apoptosis via reduction of ZEB1 abundance.^19^ In GC, miR-429 can suppress cancer cell proliferation by targeting FSCN1.^20^ Moreover, Zhu et al. found that miR-429 could trigger GC cell apoptosis by modulating Bcl-2.^21^ Our rescue assays reveal that circ_0008035 silencing suppresses GC cell malignant behaviors by increasing available miR-429 content.

Subsequently, we predicted the miR-429-mRNA interactions using the starBase tool and verified that SMAD2 is a target of miR-429. SMAD2 is a critical intracellular signal transduction mediator and transcription factor, which mediates TGF-β family signal transduction.^22^ SMAD2 can act as an oncogenic driver in human tumorigenesis.^23-25^ For example, circ_0001829 aggravates GC development by targeting the miR-155-5p/SMAD2 axis.^26^ We find that miR-429 suppresses GC progression by reducing SMAD2 expression. Importantly, we demonstrate that circ_0008035 can absorb miR-429 to enhance SMAD2 abundance, suggesting the implication of the circ_0008035/miR-429/SMAD2 axis in GC progression. Similarly, circ_0008035 has been shown to function as a promoter in GC by enhancing cancer cell growth and metastasis via miR-1256/CFACAM6 and miR-375/YBX1 axes.^14,27^ Moreover, Gao et al. reported that dexmedetomidine elevated circ_0008035 expression to contribute to GC cell ferroptosis through the miR-302a/E2F7 cascade.^28^ These findings suggest that the circ_0008035/miR-429/SMAD2, circ_0008035/miR-1256/CFACAM6, circ_0008035/miR-375/YBX1, and circ_0008035/miR-302a/E2F7 axes may form a regulatory network, which is implicated in GC development. Because 1 circRNA can target many miRNAs, there may be alternative miRNA/mRNA axes that remain to be defined in the modulation of circ_0008035 in GC. Additionally, we confirm that circ_0008035 silencing diminishes the growth of xenograft tumors in vivo. However, such in vivo data are limited by the lack of direct evidence of the novel mechanism in regulating GC cell tumorigenicity. With these findings, inhibition of circ_0008035 may be a potential strategy for GC treatment.

In conclusion, these findings unveil a novel circRNA/miRNA/mRNA cascade in regulating GC development. Circ_0008035 can facilitate GC malignant progression through mediating the miR-429/SMAD2 axis. These results may offer novel effective targets for GC therapy.

## Figures and Tables

**Table 1.** Primer Sequences Used for qPCR

Name		Primers for PCR (5’-3’)
circ_0008035	Forward	TTGTGACAAGCCCCTACCAG
	Reverse	TCCCGATAATCATACCTTGCTCT
SMAD2	Forward	GCTGGCCTGATCTTCACAGT
	Reverse	CCAGAGGCGGAAGTTCTGTT
miR-429	Forward	GCCGAGTAATACTGTCTGGTAA
	Reverse	CTCAACTGGTGTCGTGGA
β-Actin	Forward	CTTCGCGGGCGACGAT
	Reverse	CCACATAGGAATCCTTCTGACC
U6	Forward	CTCGCTTCGGCAGCACA
	Reverse	AACGCTTCACGAATTTGCGT
EXT1	Forward	ACATTCTAGCGGCCATCGAG
	Reverse	ACTCAGGACAAAGAGGCACG
